# Dietary Iron and the Elite Dancer

**DOI:** 10.3390/nu14091936

**Published:** 2022-05-05

**Authors:** Caitlin Attwell, Cory Dugan, Alannah K. A. McKay, Joanna Nicholas, Luke Hopper, Peter Peeling

**Affiliations:** 1 School of Human Sciences (Exercise and Sport Science), The University of Western Australia, Crawley, WA 6009, Australia; 22259563@student.uwa.edu.au (C.A.); cory.dugan@research.uwa.edu.au (C.D.); 2 Mary MacKillop Institute for Health Research, Australian Catholic University, Melbourne, VIC 3000, Australia; alannah.mckay@acu.edu.au; 3 Western Australian Academy of Performing Arts, Edith Cowan University, Mount Lawley, WA 6050, Australia; j.nicholas@ecu.edu.au (J.N.); l.hopper@ecu.edu.au (L.H.); 4 Western Australian Institute of Sport, Mt Claremont, WA 6010, Australia

**Keywords:** ferritin, supplement, deficiency, energy availability

## Abstract

Dancers are an athlete population at high risk of developing iron deficiency (ID). The aesthetic nature of the discipline means dancers potentially utilise dietary restriction to meet physique goals. In combination with high training demands, this means dancers are susceptible to problems related to low energy availability (LEA), which impacts nutrient intake. In the presence of LEA, ID is common because of a reduced mineral content within the low energy diet. Left untreated, ID becomes an issue that results in fatigue, reduced aerobic work capacity, and ultimately, iron deficient anaemia (IDA). Such progression can be detrimental to a dancer’s capacity given the physically demanding nature of training, rehearsal, and performances. Previous literature has focused on the manifestation and treatment of ID primarily in the context of endurance athletes; however, a dance-specific context addressing the interplay between dance training and performance, LEA and ID is essential for practitioners working in this space. By consolidating findings from identified studies of dancers and other relevant athlete groups, this review explores causal factors of ID and potential treatment strategies for dancers to optimise absorption from an oral iron supplementation regime to adequately support health and performance.

## 1. Introduction

Classical ballet and contemporary dance are performing arts disciplines that require a high degree of artistry combined with appropriate physiological fitness. As athletes, elite dancers undertake years of rigorous training in a competitive environment to reach a professional standard. They must display high levels of muscular strength, power, flexibility and skill, and are required to maintain a lean body composition. The unique aesthetic criteria create a requirement for dancers to be lean, due to the belief that this is advantageous to performance, both physically and artistically [1]. Accordingly, maintaining the desired body composition, as well as overall health and optimal physical performance, becomes a difficult balancing act. The energy demands of dance training itself are relatively low [2,3,4], with previous research reporting energy expenditures during the centre section of professional ballet class to be 4.9 kcal/min and 8.4 kcal/min for female and male dancers respectively. Comparably, mean energy expenditure during a professional contemporary dance class was 5.3 kcal/min [5]. In comparison to other activities such as running (~12–22 kcal/min, depending on running speed), these energy demands per minute of exercise are relatively low [6], meaning a longer duration is required to achieve the same level of energy expenditure. The extended duration of activity during a professional dancer’s workday (>6 h) [7] may allow the dancer to accumulate a relatively significant energy expenditure; however, due to the current aesthetic requirements favouring a very lean body composition, dancers may also utilise dietary restriction in order to further maintain such a lean physique [1]. Nonetheless, an inadequate energy intake can result in a multitude of health- and performance-related issues resulting from a state of low energy availability (LEA), which is defined as an inadequate amount of energy remaining for optimal physiological functioning after energy expended during exercise [8]. Among dancers, characteristics consistent with LEA were present in 57% of females and 29% of males, indicating the prevalence in this population [9]. Importantly, LEA from dietary restriction can increase an individual’s risk of becoming deficient in specific micronutrients, such as iron [10]. A deficiency in iron levels can be problematic as iron plays a central role in oxygen delivery, aerobic energy metabolism, and cognitive and immune functions [11]. Therefore, maintaining adequate iron stores is important for dancers whose profession relies on optimal physical performance and health [12]. Nevertheless, iron deficiency is common among athlete populations, with prevalence rates estimated to effect ~15–35% of female and ~3–11% of male athletes [13]. Comparably, the prevalence of iron deficiency of varying severity has been reported to affect 15–53% of female adolescent dancers [14,15], although iron-specific research in this population appears limited.

Alongside low energy intake, several other physiological and lifestyle factors place dancers at increased risk of iron deficiency. Dietary choices, such as adopting a vegetarian diet may limit the bioavailability of iron, resulting in decreased absorption [16]. Furthermore, female dancers may face additional iron loss due to menstrual blood loss [17]. Alongside these factors, the high volume of activity characteristic of a dancer’s training, rehearsal and performance schedules may account for further iron losses via exercise-induced avenues including haemolysis, sweating, haematuria and gastrointestinal bleeding [18,19]. Although the intensity of dance classes and rehearsals is relatively low, a professional dancer’s working day sees them completing over 6 h of activity per day with minimal breaks [7,19]. This large amount of activity may impede iron absorption during the post-exercise period due to the action of the iron regulatory hormone hepcidin, and its increased expression in response to exercise-induced inflammation [20,21], Furthermore, hepcidin has been shown to follow a pattern of diurnal variation, such that circulating levels are lowest in the morning, reaching a peak in the afternoon, meaning iron may not be absorbed as readily during this period. This creates a confined daily window for optimal iron absorption [22].

Clearly, there are several challenges that may prevent dancers from achieving a sufficient iron intake, and subsequently place them at risk of iron deficiency. It follows that increased iron intake may be necessary to counteract such deficiencies. Nutritional interventions with a focus on increasing dietary iron intake are generally the first step; however, given the low bioavailability of dietary iron sources (15–35% and 2–20%, respectively, from meat and plant-based sources), this may be insufficient to fully correct iron stores [23]. In the case of more severe deficiencies, oral iron supplementation is an effective and economical method of iron replacement, though, due to the inhibitory action of hepcidin, adoption of a strategic approach to supplement timing and dosage is required to optimise absorption and minimise any potential GI side effects that may be associated with prolonged use of oral iron tablets [24].

On account of this, the ensuing review will focus on the specific factors contributing to iron deficiency in elite classical ballet and contemporary dancers, while also examining the application of current oral iron supplementation strategies in the context of elite dancers, as a means to prevent iron deficiency in this population.

## 2. What Are the Physiological Requirements of Dance?

Dance is characterised as an intermittent form of activity, combining short bursts of high intensity movement, such as jump sequences (allegros) with more continuous, sustained bouts that require a high degree of skill and technicality, such as adagios [4,25]. Both classical ballet and contemporary dance are primarily anaerobic in nature, requiring both muscular power and endurance to execute sequences of explosive steps in a precise manner [1].

In terms of the aerobic demand of dance, previous research has indicated that ballet dancers show relatively low levels of cardiorespiratory fitness [2]. A typical ballet class generally consists of three distinct stages of increasing intensity. The first section comprises low–moderate intensity movements performed while holding the barre. Next, a series of exercises are performed in the centre of the studio at a higher intensity. This is followed by series of jumps, turns and sequences across the floor at a high intensity. All sections consist of bouts of short duration, approximately 15–60 s, followed by a longer period of rest. As intensity of the dancing increases, corresponding decreases in duration and increased rest periods arise [2]. Cardiorespiratory demands of a ballet class in terms of reported oxygen uptake during the barre section range from 36–38% of maximal oxygen uptake (VO_2max_) [2,3]. During exercises performed in the centre, oxygen uptake ranged from 43–55% of maximum, with the final stage of higher intensity allegro sequences comprising the upper end of this range [2,3]. It appears the low work–rest ratios do not allow adequate stimuli for aerobic adaptions to occur, with dance activity alone suggested to be insufficient in eliciting cardiorespiratory improvements [5]. The VO_2max_ values of advanced and professional male and female ballet dancers ranged from 37.3–57.0 mL/kg/min, and that of male and female contemporary and modern dancers ranged from 46.4–56.6 mL/kg/min [25]. Relative to other intermittent power athletes, such as soccer, squash and tennis players, these values are considered relatively low [1,26,27]. Interestingly, higher VO_2max_ and heart rate measures have been reported during rehearsals of performance material than in daily class [2,28]. It appears that there is a disparity between a dancer’s training and their performance demands, such that rehearsals and performances have a greater aerobic demand than classes. It was noted that blocks of performances brought about increases in aerobic fitness parameters of professional contemporary dancers [29]. Furthermore, professional contemporary dancers showed higher levels of aerobic fitness than pre-professional students, which is likely a reflection of their greater performing schedules [30]. For dance students, a majority of time is spent developing technique through classwork [31], whereas professional dancers, having already established a solid technical base, spend a larger proportion of their day rehearsing and performing. For example, a professional’s daily schedule generally consists of a 90 min technique class in the morning followed by several hours of rehearsal and potentially a performance in the evening [7,19]. Though largely dependent on the repertoire being presented by the company, the greater aerobic demand of performance blocks may be translated to a greater proportion of training spent at a higher intensity, resulting in a greater energy demand at the professional level.

Interestingly, there also appear to be variations in workload intensity between female classical ballet dancers of different ranks. In one professional ballet company, soloists and principal dancers spent less time at rest and more time working at a higher intensity than first artists and corps de ballet dancers [19]. Such differences can be attributed to different choreographic and rehearsal demands between the different ballet ranks. Notably, although the majority of dancers of all ranks were active for approximately 6 h/day, the average exercise intensity for these dancers remained relatively low (<4 METs), where 3–6 METs is defined as ‘moderate intensity’, and only a short time was spent at higher intensities (6–9 METs) (<5% of the day) [19]. Despite this, the energy cost of long periods of activity across the day becomes relevant in consideration of a dancer’s total daily energy expenditure, with longer durations of lower intensity activity likely accumulating a significant metabolic demand daily. However, depending on a dancer’s ability level (student or professional), ranking in a company, and the specific repertoire being rehearsed, there may be substantial variation in levels of energy expenditure between dancers.

Even so, there is the longstanding belief that being lean will enhance the aesthetics of dance performance [1]. Given this, to maintain such a lean body composition, dancers may achieve physique goals with energy restricted diet alongside energy expended from dancing. In addition, the traditional approach to dance training is highly skill-based, with less emphasis on the development of physiological fitness parameters [1,25]. This approach, combined with the full work schedules of professional dancers (involving daily classes, rehearsals of performance material and the performances themselves), provides limited opportunities for supplemental aerobic training that would increase energy expenditure and allow for aerobic adaptations to occur [19,32]. As such, in combination with their dancing/activity requirements, dancers may also turn to other methods of weight management, such as dietary restriction to meet the aesthetic criteria favoured for professional dancing.

## 3. What Are the Aesthetic and Artistic Requirements of Classical Ballet and Contemporary Dance?

Given the current belief among dance professionals that being lean will enhance the aesthetics of dance performance, it is not surprising that many pre-professional students and professional dancers exhibit a low body weight, BMI and body fat percentages [4,33,34]. In studies of female professional ballet dancers, body fat percentage ranged from 13–23% [1,7,33,34,35]. In male ballet dancers, percentage body fat ranged from 5–15% [1,33]. Often, low levels of body fat are achieved by restricting energy intake, with professional ballet dancers reported to consume less than 80% of the recommended daily allowance of energy, whereas pre-professional students consumed less than 70% [1]. During blocks of performances, an imbalance between energy intake and energy expenditure in female professional ballet dancers was shown to result in a state of energy deficit, where energy intake was insufficient to replace energy expended during exercise and at rest [7]. Similarly, a study in pre-professional female contemporary dancers found that, on average, dancers were in an energy deficit of approximately −356 kcal/day [36]. Such restrictions may lead to the breakdown of fat stores and lean body tissue, which in the case of dancers with already low levels of body fat, may cause losses in muscle mass [37]. Given the desire for leanness, and the use of dietary restriction among dancers, it is not surprising that dancers are at a significantly higher risk of suffering from an eating disorder than non-dancers. According to a 2014 meta-analysis and systematic review, the prevalence of eating disorders amongst female ballet dancers was 16.4% [38]. A similar compilation of data for male dancers is currently lacking; however, one study of 85 adolescent male dancers reported 7.6% of the cohort to be symptomatic for disordered eating [39]. Another study in male dancers described a 14% lifetime incidence of an eating disorder among male dancers [9]. The prevalence of eating disorders in female aesthetic sport athletes (from sports such as gymnastics, figure skating and diving) has been reported at 42% [40], and a recent study in male and female circus artists revealed that 36% were at risk of disordered eating [41]. Such findings highlight the frequency of unhealthy weight control practices in aesthetically judged athletic disciplines, such as dance. Ultimately, the main concern is the potential for a long-term energy deficit to cause impairments to the functioning of multiple physiological systems, which will in turn, have a vast negative impact on a dancer’s health and performance.

## 4. Low Energy Availability

Low energy availability (LEA) occurs when energy intake is insufficient to meet the energy requirements of exercise as well as maintain the physiological functions essential for optimal performance and health [8]. States of LEA can result from inadequate dietary energy intake, increased exercise energy expenditure or a combination of both [42]. A shortage of available energy can manifest in perturbed functioning of physiological processes, and may cause adverse outcomes including menstrual dysfunction, reduced bone mineral density, and metabolic disturbances such as decreased resting metabolic rate [8,43,44]. Other physiological systems potentially negatively impacted by LEA include endocrine, cardiovascular, gastrointestinal, immune, psychological and haematological functioning, as well as impairment of growth and development. The comprehensive term for the wide range of adverse effects resulting from LEA is known as ‘relative energy deficiency in sport’ or RED-S [8]. Among females, an energy availability of <30 kcal/kg/FFM/day is the accepted cut-off leading to such physiological perturbations, aside from menstrual cycle disturbances, where a specific threshold value has not been established [8,45,46]. Instead, it appears that the likelihood of menstrual cycle disturbances increases as energy availability decreases. The optimal energy availability for women is proposed to be achieved at ~45 kcal/kg/ FFM/day for healthy physiological functioning, though this value can vary depending on the individual and is not considered a universal threshold [8]. Similar gauges of energy availability in exercising men are currently lacking, despite the occurrence of RED-S in both sexes [47,48]. As such, the effects of LEA in males are poorly understood, which may reflect difficulties in identifying LEA in males due to the lack of obvious indicators such as menstrual irregularities [49].

The combination of an energy restricted diet alongside the activity of dancing, particularly during phases involving heavy rehearsal and performance components, places dancers at risk of LEA and subsequently RED-S [9]. Several studies in female aesthetic sports and performing arts have indicated that these athletes are at a high risk of developing LEA [50,51,52,53]. For instance, a study of female pre-professional ballet students found that 44% had a reduced energy availability of 30–45 kcal/kg/FFM/day, and 22% had a low energy availability of < 30 kcal/kg/FFM/day [54]. In pre-professional contemporary dancers, energy availability was similar to that reported in ballet dancers at 26 ± 13 kcal/kg/FFM/day (mean ± SD) [36]. Furthermore, assessed using the Low Energy Availability in Female Athletes Questionnaire (LEAF-Q), 65% of dancers were classified at risk of RED-S [54]. In comparison to the risk of RED-S in other female elite athletes assessed using the LEAF-Q, including Australian Football League players (30%), sprinters (39%) and cross-country runners (80%), this proportion of dancers sits at the higher end of the range [55,56,57]. It is apparent from these findings that RED-S is a concern for both ballet and contemporary dancers, though variations in time spent in class, rehearsal and performance between student and professional dancers, and between professional ranks, may result in varying degrees of exercise energy expenditure, and corresponding LEA risk.

Since LEA and RED-S can arise from an insufficient energy consumption, a specific consequence resulting from this is the potential for an inadequate micronutrient intake. In limiting their energy consumption, dancers may be reducing their opportunities to obtain an adequate intake of essential micronutrients. [10,58]. This is particularly the case for iron, as it cannot be synthesised by the body and therefore must be obtained exogenously via dietary or supplemental iron [24]. Micronutrient deficiencies, specifically iron deficiency, can have a profound effect on a dancer’s performance and overall health. Furthermore, energy expenditure may also be increased in the presence of iron deficiency, as a lack of iron can result in altered metabolic efficiency, contributing to further LEA [59]. With this in mind, the interplay between LEA and iron deficiency is of importance to dancers, as deficiencies in both energy availability and iron stores can have profound negative impact on performance and overall health.

## 5. Iron Deficiency

Iron deficiency (ID) occurs when iron levels in the body are insufficient to support physiological processes [60]. As a key component of haemoglobin and myoglobin, iron plays a pivotal role in oxygen transport to active tissues and is fundamental in the process of aerobic energy production, which is imperative to athletic endeavours such as dancing [12]. Healthy iron stores may aid dancers with recalling and executing intricate technical details and choreography, due to the role of iron in cognitive function [11]. Additionally, iron is involved in the process of mounting an appropriate immune response when faced with a pathogen, meaning adequate iron stores may reduce the number of training days lost due to illness [11].

Key causal factors in ID include a combination of inadequate dietary iron intake, increased iron losses due to exercise (and or heavy menstrual bleeding), and inhibited iron absorption due to the action of the iron regulatory hormone, hepcidin, when stimulated by exercise [18]. All the aforementioned factors are highly relevant to dancer populations, highlighting their increased risk of developing ID.

Iron deficiency ranges in severity across a cumulative spectrum, from diminished iron stores to anaemia [61]. Peeling et al. [62] categorised ID into three stages of severity:

- **Stage One—Iron Depletion:** characterised by decreased iron stores in the bone marrow, liver, and spleen (serum ferritin (sFer) < 35 µg/L, haemoglobin concentration (Hb) > 115g/L, transferring saturation (TS) > 16%).
- **Stage Two—Iron Deficient Erythropoiesis:** characterised by decreased erythrocyte production due to reduced iron supply to erythroid marrow (sFer < 20 µg/L, Hb > 115g/L, TS < 16%).
- **Stage Three—Iron Deficient Anaemia:** characterised by critically diminished iron availability and resulting reduction in haemoglobin production (sFer < 12 µg/L, Hb < 115g/L, TS < 16%).

The first two stages are known as iron deficient non-anaemia (IDNA) due to the maintenance of adequate haemoglobin levels despite a decrease in iron supply. Stage three is referred to as iron deficient anaemia (IDA); here, insufficient iron levels are unable to support haemoglobin synthesis, and thus, erythrocyte production falls, resulting in anaemia [63]. In terms of athletic performance, IDA is unequivocally detrimental, as anaemia will manifest in a reduced oxygen carrying capacity [64]. In contrast, the literature concerning the performance impacts of IDNA are conflicting, with both negative impacts and no impacts to performance being reported [63,65,66]. Reduced endurance capacity and energetic efficiency has, however, been reported as a result of IDNA [67,68,69], which may be a factor leading to early fatigue during exercise, a concern being that undue fatigue may impact a dancer’s training consistency. Additionally, if left untreated, there is the potential for dancers with IDNA to progress to a more severe iron deficiency (IDA), where reductions in oxygen transport capacity may diminish training and performance outcomes.

Serum ferritin (sFer), haemoglobin concentration (Hb) and transferrin saturation (TS) are commonly used haematological markers for the assessment of iron status [62]. However, there is a lack of clinical consensus on the exact values used to label IDNA and IDA, especially in athletes and dancers. Multiple factors influence the manifestation of ID, including sex, age, diet, exercise, pregnancy, altitude exposure, smoking and the presence of chronic disease [70,71]. Additionally, ferritin is an acute-phase protein, meaning levels increase in response to inflammation, such as during illness or in response to exercise-induced inflammation [13]. Shifts in blood plasma volume in response to training, known as ‘pseudo-anaemia’, may also affect Hb measurements [13]. These factors have made the comprehensive designation of specific values defining ID somewhat complex, hence the lack of universal consensus regarding these values.

Limited research has reported the prevalence of ID in dancer populations. A cross-sectional study by Beck et al. [14] investigating the nutritional status of 47 adolescent female ballet dancers revealed that 28% (*n* = 13) of dancers studied had suboptimal iron status (defined as sFer < 20 µg/L). Of these, four had IDNA (sFer < 12 µg/L) and one IDA (sFer < 12 µg/L, Hb < 120 g/L) [14]. Similarly, early work by Mahlamaki and Mahlamaki [15] revealed that IDA (sFer < 12 µg/L, Hb < 125 g/L) was present in 15% of female adolescent ballet and contemporary dancers studied. Low iron stores (sFer < 30 µg/L) were equally present in both dancer and in female age-matched controls (~55%) [15]. Importantly, due to differences in cut off values used, the prevalence of suboptimal iron status in these studies may have been even higher than stated, as the sFer values defined are both lower than that indicative of stage one iron depletion as defined by Peeling et al. [62] (sFer < 35 µg/L). Furthermore, both of these studies considered only female adolescent dancers. Contributing factors such as the effect of exercise, dietary iron intake and type of iron consumed (haem or hon-haem iron) did not appear to be considered as factors affecting iron status, indicating the need for further attention in future research.

No similar research was identified with male dancers; however, studies of iron status in a group of elite male athletes from a variety of sports indicated the incidence of IDNA (sFer < 20 µg/L) was 43%, demonstrating that male athletes are an at-risk population [72]. Moreover, a higher prevalence of IDNA and IDA was identified in male gymnasts compared to other non-aesthetic sport male athletes, illustrating that the risk of ID for athletes involved in these aesthetically judged disciplines may be similar between sexes [73]. Consequently, it appears that further research is warranted to identify the full extent of ID in elite dancers, particularly studies in larger populations of professional male dancers with greater consideration of factors that may influence the manifestation of ID.

## 6. Why Is ID Risk High in Dancer Populations?

The athletic nature of dance itself contributes to increased iron losses from exercise-induced avenues such as haemolysis, as dancing is highly weight bearing, as well as sweating, gastrointestinal (GI) bleeding and haematuria [18]. Notably, exercise has also been shown to cause upregulation of the iron regulatory hormone hepcidin [20,21]. Secreted by the liver, hepcidin acts by binding to the cellular iron transporter ferroportin, which controls iron absorption from the intestine, the mobilisation of stored iron from hepatocytes, and the recycling of iron from senescent erythrocytes [74]. The upregulation of hepcidin causes internalisation and degradation of ferroportin, effectively inhibiting iron absorption and recycling [75]. Hepcidin release is under homeostatic regulation, driven by iron levels in the body. In times of low iron stores, anaemia, or hypoxia, hepcidin release is inhibited to increase iron availability necessary for the maintenance of physiological processes [76]. In contrast, hepcidin release is stimulated during periods of iron loading [77] or during infection and illness in order to sequester iron stores and prevent pathogen propagation [78]. This control mechanism of iron in the body is important since excess iron can be detrimental due to its ability to react with oxygen and generate radical oxygen species, leading to tissue damage [79]. Accordingly, hepcidin acts as a protective mechanism against excessive iron accumulation, although in the exercise context this mechanism presents an additional challenge to dancers, given its potential to exacerbate the impact of net iron loss or insufficient dietary iron intake.

## 7. Hepcidin, the Inflammatory Response, and Exercise (Dancing)

Over the last decade, a significant amount of research has shown hepcidin synthesis increases in response to exercise [20,21,80,81,82]. Interestingly, the inflammatory cytokine interleukin-6 (IL-6) released in response to inflammation or infection causes the upregulation of hepcidin [83], and the inflammatory nature of exercise has been shown to elicit a similar response. Previously, Peeling and colleagues [20] concluded that the IL-6 response to exercise was associated with an increase in hepcidin concentrations in the 3–6-h period post exercise [20]. Similar findings were reported by Newlin et al. [21], who expanded this work to suggest that exercise duration influences the hepcidin response, reporting a two-times-greater increase in hepcidin levels after 120 min of running, as compared to a 60 min, intensity-matched equivalent [21]. Further work by Peeling et al. [84] added that an athlete’s underlying iron stores may also dictate the magnitude of the hepcidin response to exercise, where low iron stores suppress hepcidin release, which appears to override any inflammatory-driven increases [84]. Recent findings indicate increased iron absorption when ferritin levels are <51 μg/L, which corresponds to hepcidin levels < 3 nm/L [85]; a potential response to the body’s need for additional iron when presenting as ID. Interestingly, recent research in military personnel has indicated that energy deficiency may exacerbate the hepcidin response to physical activity, such that post-exercise hepcidin levels were 108% greater, and dietary iron absorption was lower for those in a state of energy deficit, in comparison to those in an energy balanced state, despite comparable increases in IL-6 [82]. Furthermore, studies in endurance athletes consuming a low carbohydrate diet for 1–6 days saw a greater post-exercise IL-6 response and post-exercise hepcidin levels than those consuming a high carbohydrate diet [86,87]. Such findings are highly relevant for dancers, given the prevalence of dietary restriction and chronic LEA where both energy and thus carbohydrate consumption may be insufficient. This may in fact exacerbate the post exercise hepcidin response, creating another barrier to adequate iron absorption.

The nature of professional dancers’ schedules sees them performing multiple bouts of activity throughout the day with minimal breaks [19,32]. Recent findings for the effect of twice a day training on the hepcidin response, have suggested that hepcidin levels are not augmented by further exercise, and instead remain consistently high [88], meaning dancers are likely to face elevated hepcidin levels in any rehearsal or performances following initial classes or activities earlier in the day. Since the magnitude of the hepcidin response is also governed by exercise duration, it is possible that dancers face a significant degree of exercise-induced inflammation with subsequent increases in hepcidin, due to the multiple, prolonged sessions across the day. To date, there have been no studies investigating the hepcidin response in dancers, presenting as an interesting area for future exploration.

The above research addressed the exercise-induced hepcidin response to endurance exercise. As previously discussed, classical ballet and contemporary dance are predominately anaerobic, intermittent forms of activity [4]. Research has suggested that exercise modality does not affect the hepcidin response; however, the degree of haemolysis from weight-bearing exercise may influence iron losses in the long term [89]. Dance is a high impact, weight-bearing activity meaning dancers may experience a significant degree of haemolysis from repeated jumps and landings, potentially contributing to increased iron losses in the long term [90,91]. Accordingly, the compromised iron absorption and increased iron losses that arise from the activity of dancing itself place dancers at risk of developing ID. Therefore, dancers need to take particular care during blocks of increased training, rehearsals and or performances, where activity levels are high and iron status is at risk of becoming diminished.

## 8. Hepcidin Diurnal Variation

Alongside the inflammation-induced changes in hepcidin levels, it has also been well established that hepcidin shows a cycle of diurnal variation, whereby hepcidin levels are lowest in the morning and reach a peak in the afternoon [22,92,93,94,95]. Findings by Kemna et al. [22] reported a 2–6-fold increase in serum hepcidin concentration from 06:00 a.m. to 15:00 p.m. on the same day [22]. Thus, variations in hepcidin levels over the course of the day ultimately prompt corresponding changes in iron absorption. Investigation of this effect in a study involving endurance-trained runners with suboptimal iron status (sFer < 50 µg/L) examined the outcomes of morning and afternoon exercise on iron absorption [80]. It was found that iron was best absorbed in the morning, following exercise. A larger hepcidin response was seen in the afternoon following exercise, which was suggested to be the combined effect of exercise-induced inflammation and the diurnal effect of hepcidin. This was associated with a lower degree of iron absorption from a meal consumed post exercise in the afternoon. Additionally, resting levels of hepcidin in the afternoon trial were seen to increase threefold from 06:00 a.m. to 15:30 p.m. in the absence of exercise [80].

This short-term study provides insight into the potential effect of the diurnal nature of hepcidin in an athletic population, though long-term studies of this effect, and the impact on training scheduling are yet to occur. However, consideration of this diurnal variation is of importance to dancers, as iron consumed later in the day may not be absorbed as readily when compared to the morning, prior to the diurnal increase. This potentially narrow window for optimal iron absorption presents an additional challenge for dancers aiming to achieve sufficient iron intake.

## 9. Menstrual Blood Loss

Among female dancers, iron loss due to menstruation is another component potentially contributing to ID. Menstrual blood loss can contribute to iron losses of approximately 6.4–11.2 mg per menses [96]. Accordingly, each 1 mg/day increase in menstrual iron loss can result in a 6 × 9 μg/L decrease in serum ferritin levels [96]. Furthermore, a significant negative association is known to exist between estimated monthly menstrual blood loss and serum ferritin concentrations in female collegiate level athletes, meaning lower iron stores in athletes may be attributed in part to greater blood losses during the menses [97]. Individuals experiencing heavy menstrual bleeding (HMB), defined as excessive menstrual blood loss (≥80 mL per menstrual cycle) are at a higher risk of iron deficiency [17,98]. A survey conducted in active females found that, 54% of exercising females and 37% of elite female athletes experienced HMB. These findings were associated with self-reported anaemia and increased use of iron supplements [99]. Accordingly, a recent study found that women with HMB were more likely to report a history of ID (51.18%) compared to those without HMB (37.58%), and a significant correlation (*p* < 0.001) was found between HMB and self-reported symptoms relating to anaemia. These findings emphasise the heightened ID risk of exercising with HMB [100]. Such observations suggest that iron loss due to menstruation is a primary reason accounting for the 10–15% greater prevalence of ID in exercising women compared to exercising men [101]. Therefore, female dancers with eumenorrheic cycles, defined as regularly occurring menstruation every 24–35 days, may be at a greater risk of ID due to increased iron losses via menstrual blood loss [102,103].

Alternatively, menstrual dysfunction such as oligomenorrhea and amenorrhea is also a concern for aesthetic athletes and dancers [104,105]. Oligomenorrhea is the occurrence of irregularities in menstrual cycle length, and amenorrhea is defined as an absence of menses for at least 90 days [102,103]. A study in deployed female military personnel found that individuals with amenorrhea displayed higher serum ferritin levels than those who were menstruating, such that the increased iron loss from menses further compromised iron status [106]. Menstrual dysfunction is an outcome of RED-S, thus is brought about by states of chronic LEA [8,45,46]. Accordingly, menstrual dysfunction was reported in a higher percentage of athletes participating in weight-sensitive sports (including judo, karate and wrestling), and sports that emphasised leanness (rhythmic gymnastics, figure skating and diving) (24.8%), as compared to those involved in other sports where body composition is less of a concern, such as basketball, tennis and golf (13.1%) [104]. Furthermore, menstrual dysfunction was reported in 40% of female vocational ballet students, where 65% of this cohort were classified at risk of RED-S [54]. Such outcomes highlight the link between LEA and RED-S associated issues with menstruation, such as amenorrhea or oligomenorrhea.

Interestingly, the presence of severe menstrual disorders commonly associated with LEA, appear to be protective against iron deficiency [8,103]. Although indicative of an underlying energy deficiency, irregular or absent menses may have the surrogate effect of preventing further iron losses [103]. The issue, however, is circular, since it is plausible that dancers experiencing menstrual dysfunction from LEA due to dietary restriction are already at risk of a compromised iron status as a function of inadequate iron intake [58]. Therefore, the absence of menses in female dancers should not be considered beneficial, or an indication of optimal health. As such, dancers with eumenorrheic menstrual cycles should be aware of the extra iron losses that accompany menses, especially if experiencing HMB. Conversely, those with amenorrhea or oligomenorrhea may benefit from consultation with a medical professional to assess the underlying cause of the dysfunction, such as LEA.

## 10. Vegetarian/Vegan Diets and ID Conjuncture

Dancers who follow a vegan or vegetarian diet face a further challenge to their iron stores, primarily due to the low bioavailability of iron from plant-based sources. Dietary iron is present in two forms, haem and non-haem iron, with a bioavailability of 15–35% and 2–20%, respectively [23]. The absorption of non-haem iron, from both plant and animal sources, is influenced by several dietary inhibitors, and therefore, is a less resourceful iron source. Polyphenols, tannins (found in tea and coffee), calcium, and phytates (found in wholegrains, nuts, and legumes) are the main inhibitors that interact with non-haem iron and hinder its absorption [13]. Conversely, vitamin C is a promoter of non-haem iron absorption, acting by converting it into a more soluble ferrous form, where the consumption of 50–75 mg of ascorbic acid (vitamin C) associated with a three- to four-fold increase in the absorption of non-haem iron [23,107]. In comparison to non-haem iron, haem iron, found in the haemoglobin and myoglobin of meat sources, is not affected by these dietary enhancers or inhibitors, hence is absorbed more readily [23]. Consequently, haem iron may provide up to one third of the dietary iron absorbed, despite making up only 10% of dietary iron consumed [64]. A 2018 meta-analysis and systematic review of the effect of vegetarian or vegan diets on the iron status of adults found that those adopting plant-based diets had lower iron stores (sFer levels) than those consuming diets that incorporated animal-based products [108]. Since vegetarians rely primarily on non-haem iron sources, the absorption inefficiencies clearly explain the increased risk of ID [24].

Dancers may adopt a vegetarian diet due to the belief that it is an effective means of weight management, or for ethical, religious or health reasons [109]. A recent study reporting indicators and correlates of LEA in dancers found that 23% of female and 5% of male advanced level dancers were vegetarian, whilst 10% of male and female dancers were vegan [9]. It should be noted, however, that female participants in this study outnumbered males by over tenfold, thus, findings may not clearly represent the male dancer population. Furthermore, 28% of pre-professional contemporary dancers reported being vegetarian, vegan or avoided red meat [36]. These findings highlight the occurrence of dancers relying predominately on inefficient, non-haem iron sources, potentially creating a barrier to the maintenance of adequate iron stores.

Diets that restrict certain food groups can also increase the risk of both LEA and associated nutrient deficiencies [110]. Energy intake among vegetarians is typically lower than non-vegetarians [16,111]. This may be due to the high fibre content of plant-based foods causing increased levels of satiety, leading to a decreased energy intake [110]. A properly planned vegetarian or vegan diet can provide adequate levels of energy and essential nutrients such as iron; however, in poorly planned and or energy-restricted vegetarian/vegan diets, nutrient deficiencies may arise [112]. In short, it is the combination of the reduced bioavailability of non-haem iron and the adoption of an improperly planned or energy restricted diet which may contribute to the increased risk of ID among dancers adopting vegetarian or vegan diets. Accordingly, dancers choosing such approaches to food should work with a trained sports dietician to ensure they are achieving their required energy and nutrient intake goals.

## 11. What Are the Current Treatment Strategies for ID?

In order to correct ID, an increased external supply of iron is required as the body cannot endogenously synthesise iron [24]. When anaemia isn’t present, increasing dietary iron intake is generally the initial approach to correcting iron status [13]. In the case of dancers in a state of LEA, this is a positive first step, as increasing overall energy intake may negate the effects of other nutrient deficiencies and negative physiological outcomes associated with LEA [113].

There is limited research assessing the effects of dietary changes on iron status in dancers, though, a small study has been conducted in rhythmic gymnasts, this being a discipline with similar aesthetic requirements to dance. This study assessed the outcomes of a four-week nutritional intervention of an iron rich diet in eight collegiate level rhythmic gymnasts. Here, participants consumed a diet consisting of 15 mg of iron per day, which resulted in significantly higher sFer levels. While iron status improved in these gymnasts, no corrections in anaemia were identified [114]. This may be due to the intervention period of 4 weeks being insufficient to allow for any notable change in haemoglobin status. Additionally, given the low bioavailability of iron, 15 mg per day may have been insufficient to allow for major corrections in iron status, especially since the recommended daily iron intake (RDI) for pre-menopausal adult women is 18 mg/day [115]. Furthermore, daily iron requirements for athletic populations may even be higher given the exercise-related barriers to iron absorption. Other studies administering supplemental iron utilised much higher doses of 100–200 mg of elemental iron per day to elicit improvements in serum ferritin [61,116,117], suggesting that such a low dose is not likely to have a profound effect on iron stores. In fact, a higher iron intake results in a higher absolute absorption, despite decreases in fractional absorption [77]. Other limitations to this study include the small sample size and lack of separate control group used as a comparator. Additionally, the proportions of haem and non-haem iron consumed were not specified, making it hard to estimate the potential for iron absorption over the study period. Clearly, further research is needed to ascertain the effects of increasing dietary intake of iron in dancer and aesthetic athlete populations.

The low bioavailability of iron and the interaction of hepcidin, combined with the increased iron demand in dancer populations suggests that dietary intervention alone may be insufficient to fully correct ID, especially if the dancer is vegetarian/vegan, experiencing menstrual blood loss, or in the case of severe deficiency such as IDA. Therefore, beyond dietary intervention, oral iron therapy could be an effective strategy for iron replacement. Oral iron supplements come in many forms, with ferrous salts, specifically ferrous sulphate, being the most common, effective and economical preparation [118]. Several other tablet or liquid forms of iron supplement exist, varying in bioavailability, dosage, chemical form (ferric or ferrous), and galenic form (fast or slow release), each with varied responses and side effects [24]. Oral iron supplementation consisting of 105 mg of elemental iron in IDNA female athletes has been shown to improve sFer levels from baseline by an average of ~14 μg/L over a 28-day period [61]. Furthermore, several studies of longer duration (6–12 weeks) in exercising populations have highlighted the effectiveness of oral iron supplementation in increasing serum ferritin levels from baseline by 33–127% [119,120,121,122,123].

Although ID appears to be a significant issue among the dancer population, minimal research currently exists ascertaining the efficacy of oral iron supplementation in this population. Mahlamäki and Mahlamäki [15], is one of the only published studies to investigate the effect of oral iron supplementation on dancers’ sFer and haemoglobin levels. In this study, a sample of 25 adolescent ballet and contemporary dancers and 23 non-dancer controls were provided a daily oral iron supplement of ferrous sulphate consisting of 100 mg of elemental iron if low serum ferritin (sFer < 30 µg/L) levels were detected. These researchers described a reduction in the number of anaemic adolescent females (both dancer and controls) following 10 weeks of iron supplementation; however, no change was seen in those with reduced iron stores (i.e., IDNA) [15]. Many factors including diet, the diurnal variation of hepcidin and exercise-induced inflammation may have played a role here; however, activity levels and the time of day at which the iron supplement was taken are unspecified. Additionally, no dietary data was recorded, and therefore, the effects of dietary inhibitors/enhancers and the contribution of haem and non-haem iron to overall iron intake cannot be ascertained. Given these results we can conclude that similar to the general population, oral iron supplementation was useful in the prevention of dancers becoming anaemic. However, there is a need for updated research addressing oral iron supplementation in dancers that takes the numerous factors unexplored into account.

For oral supplementation to be most effective, prolonged adherence of ~4–12 weeks is required [61,69,119,120]. However, prolonged use of oral iron supplements has been associated with adverse effects such as gastro-intestinal (GI) discomfort, nausea, vomiting, diarrhoea and constipation. These adverse effects may lead to non-compliance with the supplementation protocol, thereby limiting the effectiveness of this approach [118,124]. To support compliance with required supplementation protocols, several strategies to minimise side effects, while optimising iron absorption have been investigated, for example, manipulating the dosage of elemental iron administered in an oral supplement.

Though it is currently unclear whether the GI symptoms manifest in a dose-dependent manner [24], existing research does show that IDA women who supplemented with 100 mg of elemental iron had a (non-significant) 40% lower occurrence rate of GI side effects in comparison to consuming a 200 mg dose [116]. Such findings indicate that a lower dose of iron is potentially beneficial in terms of increasing tolerability. Interestingly, lowering the amount of iron consumed per dose also appears to maximise fractional absorption. For instance, a study of IDNA women indicated that oral iron doses ≥ 60 mg caused an increase in hepcidin up to 24 h later, and a subsequent reduction in fractional absorption by 35–45%. Furthermore, despite a sixfold increase in iron dose, absolute iron absorption only increased threefold [77]. Overall, the absolute absorption of iron is greater with a higher iron intake; however, fractional absorption per dose is enhanced at a lower dose. Since the tolerability of a supplement is potentially increased with a lower dose, the best approach would be an individualised one that weighs up the benefits of increased iron absorption against the costs of GI symptoms.

As considered previously, the increase in hepcidin following iron consumption is a biological safeguard to prevent iron overload, or in the case of increases in the presence of inflammation, to withhold iron from the potential threat of invading pathogens [11]. However, the inflammatory response to exercise is clearly a transient prospect, and therefore, this protective innate mechanism of iron regulation may become an issue for improving iron uptake in active populations. Thus, iron supplementation strategies that reduce the magnitude of the hepcidin response to an iron load may be beneficial for active populations who are already potentially facing increased hepcidin levels from inflammation, as a method of aiding iron absorption.

With the aim of reducing the iron load per dose, and thus, the magnitude of the hepcidin response, a split-dose strategy has been investigated in iron deficient women (sFer ≤ 25 µg/L) [125]. However, it was found that splitting a single oral dose of 120 mg of iron into two 60 mg iron doses taken in the morning and afternoon did not improve iron absorption. In fact, the twice-daily divided dose group saw higher serum hepcidin levels, suggesting that the iron load from the morning dose caused an increase in hepcidin levels and the consequent local suppression of intestinal iron uptake from the second dose consumed in the afternoon [125]. Similar findings were seen in a short-term study by Moretti et al. [77], where a 60 mg dose of iron in the morning had a marked suppression effect on the fractional absorption of iron from subsequent doses in the afternoon and the following morning, suggesting the split-dose technique does not provide additional benefit beyond morning-only supplementation [77]. Furthermore, this strategy has been applied to an athletic population during a period of extended altitude exposure [126]. Athletes taking a single 200 mg dose of iron daily saw a greater increase in Hb mass than those taking a split quantity of two 100 mg doses daily. While GI symptoms appeared to be less pronounced in the split dose group, negative GI symptoms in the single dose group subsided by the third week of the intervention. These outcomes suggest that a lower dose (as seen by the 100 mg split dose) may improve tolerability, though adaptations to the tolerability of a larger dose appear to occur after two weeks of supplementation. In a practical sense, these findings attest to the inefficiency of the split-dose strategy in improving iron status; however, it appears that a gradual increase in supplement dosage over the first two weeks of supplementation is beneficial in improving tolerability while also improving iron stores [126].

Accordingly, given the efficacy of a lower dose of iron in minimising GI symptoms, other strategies to reduce the acute iron load per dose have also been considered. The implementation of a larger window between iron doses is one such strategy, specifically, supplementation on alternate days instead of daily. Stoffel and colleagues found that supplementing with a single dose of 60 mg of iron on alternate days (for 28 days) notably increased fractional and absolute iron absorption, compared with dosing iron every day for 14 days [125]. These findings were corroborated by a more recent study using higher doses, where supplementing with 200 mg of iron on alternate days resulted in twice the total absorption of the 100 mg dose administered daily [116]. Application of the alternate day strategy has also been assessed in an athlete population with suboptimal iron (sFer < 50 µg/L) [117]. Results of this study showed that both the daily and alternate day supplement strategies increased sFer by ~60% in endurance trained runners; however, the alternate day approach was associated with lower levels of GI upset, which may facilitate athletes’ adherence to a long-term supplementation regime. The alternate day strategy is supported by growing research indicating it results in reduced likelihood of negative GI symptoms and increased fractional absorption of iron [77,116,117,125]. Given the effectiveness of the alternate day approach, it is clear that changes in hepcidin levels play a significant role in the iron absorption of individuals. Therefore, for ID dancers, it is crucial to consider all methods that will potentially maximise iron absorption from any iron supplementation strategy, including accounting for the diurnal nature of hepcidin, and for the prospects of alternate day supplementation to increase fractional absorption and consistency in commitment to the supplement regime.

## 12. Summary and Conclusions

The maintenance of adequate iron levels is crucial for elite dancers to avoid detriments to overall health and performance. Nevertheless, it remains a challenge for dancers to retain an adequate iron status, due to the combination of several exercise- and dance-specific factors that place them at an increased risk of developing iron deficiency. Supplementation with oral iron tablets is a simple and cost-effective method to improve iron stores; however, a strategic approach to the timing of supplementation is required to ensure optimal absorption. In applying the research and strategies discussed in this review to the context of dancers, it is clear that dancers are a population at risk of developing ID. Furthermore, those with suboptimal iron status may benefit from an oral iron supplement to increase iron stores. Current research findings suggest that, for optimal absorption, supplementation with iron in the morning prior to exercise, may be recommended. For those experiencing GI sensitivity, supplementing on alternate days rather than daily appears effective in reducing the side effects associated with iron supplementation. It is also likely that this approach will reduce the dietary iron load, allowing for an increased fractional iron absorption. It is recommended that dancers avoid the co-consumption of foods and beverages containing polyphenols, tannins, phytates and calcium, such as tea, coffee, wholegrain cereals and dairy, within 60 min of iron supplementation, as these foods will inhibit iron absorption. Taking iron in combination with a source of vitamin C (50–75 mg) is encouraged due to its enhancing effect on iron absorption. Vegetarian dancers need to take particular care when planning their diet due to the reliance on less-well-absorbed non-haem iron sources. Dancers in a state of LEA may consider increasing their energy intake to increase availability of micronutrients such as iron in the diet, and also negate the several other health and performance effects of chronic LEA. In order to effectively manage these potential dietary strategies, dancers should work with a trained sports dietician for optimal outcomes.

## References

[1] Koutedakis Y., Jamurtas A. (2004). The Dancer as a Performing Athlete: Physiological Considerations. Sports Med..

[2] Schantz P., Åstrand P.O. (1984). Physiological characteristics of classical ballet. Med. Sci. Sports Exerc..

[3] Cohen J.L., Segal K.R., Witriol I., McArdle W.D. (1982). Cardiorespiratory responses to ballet exercise and the VO2max of elite ballet dancers. Med. Sci. Sports Exerc..

[4] Twitchett E.A., Koutedakis Y., Wyon M.A. (2009). Physiological Fitness and Professional Classical Ballet Performance: A Brief Review. J. Strength Cond. Res..

[5] Beck S., Redding E., Wyon M.A. (2015). Methodological considerations for documenting the energy demand of dance activity: A review. Front. Psychol..

[6] Koehler K., de Marees M., Braun H., Schaenzer W. (2013). Evaluation of two portable sensors for energy expenditure assessment during high-intensity running. Eur. J. Sport Sci..

[7] Kim S.Y., Cho J.H., Lee J.H., Jung J.H. (2019). Changes in Body Composition, Energy Metabolism, and Appetite-Regulating Hormones in Korean Professional Female Ballet Dancers Before and After Ballet Performance. J. Danc. Med. Sci..

[8] Mountjoy M., Sundgot-Borgen J.K., Burke L.M., Ackerman K.E., Blauwet C., Constantini N., Lebrun C., Lundy B., Melin A., Meyer N. (2018). IOC consensus statement on relative energy deficiency in sport (RED-S): 2018 update. Br. J. Sports Med..

[9] Keay N., Overseas A., Francis G. (2020). Indicators and correlates of low energy availability in male and female dancers. BMJ Open Sport Exerc. Med..

[10] Sundgot-Borgen J., Garthe I. (2011). Elite athletes in aesthetic and Olympic weight-class sports and the challenge of body weight and body compositions. J. Sports Sci..

[11] Beard J.L. (2001). Iron biology in immune function, muscle metabolism and neuronal functioning. J. Nutr..

[12] Williams M.H. (2005). Dietary Supplements and Sports Performance: Minerals. J. Int. Soc. Sports Nutr..

[13] Sim M., Garvican-Lewis L.A., Cox G.R., Govus A., McKay A.K.A., Stellingwerff T., Peeling P. (2019). Iron considerations for the athlete: A narrative review. Eur. J. Appl. Physiol..

[14] Beck K.L., Mitchell S., Foskett A., Conlon C.A., Von Hurst P.R. (2015). Dietary intake, anthropometric characteristics, and iron and Vitamin D status of female adolescent ballet dancers living in New Zealand. Int. J. Sport Nutr. Exerc. Metabol..

[15] Mahlamaki E., Mahlamaki S. (1988). Iron deficiency in adolescent female dancers. Br. J. Sports Med..

[16] Venderley A.M., Campbell W.W. (2006). Vegetarian Diets: Nutritional Considerations for Athletes. Sports Med..

[17] Pedlar C.R., Brugnara C., Bruinvels G., Burden R. (2018). Iron balance and iron supplementation for the female athlete: A practical approach. Eur. J. Sport Sci..

[18] Peeling P., Dawson B., Goodman C., Landers G., Trinder D. (2008). Athletic induced iron deficiency: New insights into the role of inflammation, cytokines and hormones. Eur. J. Appl. Physiol..

[19] Twitchett E., Angioi M., Koutedakis Y., Wyon M. (2010). The demands of a working day among female professional ballet dancers. J. Danc. Med. Sci..

[20] Peeling P., Dawson B., Goodman C., Landers G., Wiegerinck E.T., Swinkels D.W., Trinder D. (2009). Effects of exercise on hepcidin response and iron metabolism during recovery. Int. J. Sport Nutr. Exerc. Metabol..

[21] Newlin M.K., Williams S., McNamara T., Tjalsma H., Swinkels D.W., Haymes E.M. (2012). The effects of acute exercise bouts on hepcidin in women. Int. J. Sport Nutr. Exerc. Metabol..

[22] Kemna E.H.J.M., Tjalsma H., Podust V.N., Swinkels D.W. (2007). Mass Spectrometry-Based Hepcidin Measurements in Serum and Urine: Analytical Aspects and Clinical Implications. Clin. Chem..

[23] Craig W.J. (1994). Iron status of vegetarians. Am. J. Clin. Nutr..

[24] McCormick R., Sim M., Dawson B., Peeling P. (2020). Refining Treatment Strategies for Iron Deficient Athletes. Sports Med..

[25] Rodrigues-Krause J., Krause M., Reischak-Oliveira Á. (2015). Cardiorespiratory Considerations in Dance: From Classes to Performances. J. Danc. Med. Sci..

[26] Manari D., Manara M., Zurini A., Tortorella G., Vaccarezza M., Prandelli N., Ancelotti D., Vitale M., Mirandola P., Galli D. (2016). VO2Max and VO2AT: Athletic performance and field role of elite soccer players. Sport Sci. Health.

[27] Smekal G., Von Duvillard S.P., Rihacek C., Pokan R., Hofmann P., Baron R., Tschan H., Bachl N. (2001). A physiological profile of tennis match play. Med. Sci. Sports Exerc..

[28] Rodrigues-Krause J., Krause M., Cunha G.D.S., Perin D., Martins J.B., Alberton C.L., Schaun M.I., De Bittencourt P.I.H., Reischak-Oliveira A. (2014). Ballet dancers cardiorespiratory, oxidative and muscle damage responses to classes and rehearsals. Eur. J. Sport Sci..

[29] Wyon M.A., Redding E. (2005). Physiological Monitoring of Cardiorespiratory Adaptations During Rehersal and Performance of Contemporary Dance. J. Strength Cond. Res..

[30] Bronner S., Codman E., Hash-Campbell D., Ojofeitimi S. (2016). Differences in Preseason Aerobic Fitness Screening in Professional and Pre-professional Modern Dancers. J. Danc. Med. Sci..

[31] Volkova V.G., Black A.M., Kenny S.J. (2020). Internal Training Load Measures in Elite Adolescent Ballet Dancers. J. Danc. Med. Sci..

[32] Kozai A.C., Twitchett E., Morgan S., Wyon M.A. (2020). Workload Intensity and Rest Periods in Professional Ballet: Connotations for Injury. Int. J. Sports Med..

[33] Liiv H., Wyon M.A., Jürimäe T., Saar M., Mäestu J., Jürimäe J. (2013). Anthropometry, somatotypes, and aerobic power in ballet, contemporary dance, and DanceSport. Med. Probl. Perform. Art..

[34] Gammone M.A., D’Orazio N. (2020). Assessment of body composition and nutritional risks in young ballet dancers—The bioelectrical impedance analysis. J. Electr. Bioimped..

[35] Doyle-Lucas A.F., Akers J.D., Davy B.M. (2010). Energetic efficiency, menstrual irregularity, and bone mineral density in elite professional female ballet dancers. J. Danc. Med. Sci..

[36] Brown M.A., Howatson G., Quin E., Redding E., Stevenson E.J. (2017). Energy intake and energy expenditure of pre-professional female contemporary dancers. PLoS ONE.

[37] El Ghoch M., Soave F., Calugi S., Dalle Grave R. (2013). Eating disorders, physical fitness and sport performance: A systematic review. Nutrients.

[38] Arcelus J., Witcomb G.L., Mitchell A. (2014). Prevalence of Eating Disorders amongst Dancers: A Systemic Review and Meta-Analysis: Eating Disorders and Dance. Eur. Eat. Disord. Rev..

[39] Nordin-Bates S.M., Walker I.J., Redding E. (2011). Correlates of Disordered Eating Attitudes Among Male and Female Young Talented Dancers: Findings From the UK Centres for Advanced Training. Eat. Dis..

[40] Sundgot-Borgen J., Torstveit M.K. (2004). Prevalence of Eating Disorders in Elite Athletes Is Higher Than in the General Population. Clin. J. Sport Med..

[41] Van Rens F.E., Metse A.P., Heritage B. (2022). Exploring the mental health of circus artists: Circus factors, psychological resilience, and demographics predict disordered eating and exercise addictions. Psychol. Sport Exerc..

[42] Keay N., Francis G. (2019). Infographic. Energy availability: Concept, control and consequences in relative energy deficiency in sport (RED-S). Br. J. Sports Med..

[43] Nattiv A., Loucks A.B., Manore M.M., Sanborn C.F., Sundgot-Borgen J., Warren M.P. (2007). American College of Sports Medicine position stand. The female athlete triad. Med. Sci. Sports Exerc..

[44] Koehler K., De Souza M.J., Williams N.I. (2017). Less-than-expected weight loss in normal-weight women undergoing caloric restriction and exercise is accompanied by preservation of fat-free mass and metabolic adaptations. Eur. J. Clin. Nutr..

[45] Lieberman J.L., De Souza M.J., Wagstaff D.A., Williams N.I. (2018). Menstrual disruption with exercise is not linked to an energy availability threshold. Med. Sci. Sports Exerc..

[46] Williams N.I., Leidy H.J., Hill B.R., Lieberman J.L., Legro R.S., De Souza M.J. (2015). Magnitude of daily energy deficit predicts frequency but not severity of menstrual disturbances associated with exercise and caloric restriction. Am. J. Physiol. Endocrinol. Metabol..

[47] Logue D., Madigan S.M., Delahunt E., Heinen M., McDonnell S.J., Corish C.A. (2018). Low Energy Availability in Athletes: A Review of Prevalence, Dietary Patterns, Physiological Health, and Sports Performance. Sports Med..

[48] Schofield K.L., Thorpe H., Sims S.T. (2020). Where are all the men? Low energy availability in male cyclists: A review. Eur. J. Sport Sci..

[49] Slater J., Brown R., McLay-Cooke R., Black K. (2017). Low Energy Availability in Exercising Women: Historical Perspectives and Future Directions. Sports Med..

[50] Alwan N., Moss S.L., Elliott-Sale K.J., Davies I.G., Enright K. (2019). A narrative review on female physique athletes: The physiological and psychological implications of weight management practices. Int. J. Sport Nutr. Exerc. Metabol..

[51] Meng K., Qiu J., Benardot D., Carr A., Yi L., Wang J., Liang Y. (2020). The risk of low energy availability in Chinese elite and recreational female aesthetic sports athletes. J. Int. Soc. Sports Nutr..

[52] Torres-McGehee T.M., Emerson D.M., Pritchett K., Moore E.M., Smith A.B., Uriegas N.A. (2020). Energy Availability with or without Eating Disorder Risk in Collegiate Female Athletes and Performing Artists. J. Athl. Train..

[53] Villa M., Villa-Vicente J.G., Seco-Calvo J., Mielgo-Ayuso J., Collado P.S. (2021). Body Composition, Dietary Intake and the Risk of Low Energy Availability in Elite-Level Competitive Rhythmic Gymnasts. Nutrients.

[54] Civil R., Lamb A., Loosmore D., Ross L., Livingstone K., Strachan F., Dick J.R., Stevenson E.J., Brown M.A., Witard O.C. (2019). Assessment of dietary intake, energy status, and factors associated with RED-S in vocational female ballet students. Front. Nutr..

[55] Sygo J., Coates A.M., Sesbreno E., Mountjoy M.L., Burr J.F. (2018). Prevalence of indicators of low energy availability in elite female sprinters. Int. J. Sport Nutr. Exerc. Metabol..

[56] Jesus F., Castela I., Silva A.M., Branco P.A., Sousa M. (2021). Risk of low energy availability among female and male elite runners competing at the 26th European cross-country championships. Nutrients.

[57] Condo D., Lohman R., Kelly M., Carr A. (2019). Nutritional intake, sports nutrition knowledge and energy availability in female Australian rules football players. Nutrients.

[58] Sousa M., Carvalho P., Moreira P., Teixeira V.H. (2013). Nutrition and nutritional issues for dancers. Med. Probl. Perform. Art..

[59] Petkus D.L., Murray-Kolb L.E., De Souza M.J. (2017). The Unexplored Crossroads of the Female Athlete Triad and Iron Deficiency: A Narrative Review. Sports Med..

[60] Zimmermann M.B.D., Hurrell R.F.P. (2007). Nutritional iron deficiency. Lancet.

[61] Dawson B., Goodman C., Blee T., Claydon G., Peeling P., Beilby J., Prins A. (2006). Iron supplementation: Oral tablets versus intramuscular injection. Int. J. Sport Nutr. Exerc. Metabol..

[62] Peeling P., Blee T., Goodman C., Dawson B., Claydon G., Beilby J., Prins A. (2007). Effect of iron injections on aerobic-exercise performance of iron-depleted female athletes. Int. J. Sport Nutr. Exerc. Metabol..

[63] Burden R.J., Morton K., Richards T., Whyte G.P., Pedlar C.R. (2015). Is iron treatment beneficial in, iron-deficient but non-anaemic (IDNA) endurance athletes? A systematic review and meta-analysis. Br. J. Sports Med..

[64] Beard J., Tobin B. (2000). Iron status and exercise. Am. J. Clin. Nutr..

[65] Rubeor A., Goojha C., Manning J., White J. (2018). Does Iron Supplementation Improve Performance in Iron-Deficient Nonanemic Athletes?. Sports Health.

[66] Houston B.L., Hurrie D., Graham J., Perija B., Rimmer E., Rabbani R., Bernstein C.N., Turgeon A.F., Fergusson D., Houston D.S. (2018). Efficacy of iron supplementation on fatigue and physical capacity in non-anaemic iron-deficient adults: A systematic review of randomised controlled trials. BMJ Open.

[67] Brownlie T., Utermohlen V., Hinton P.S., Haas J.D. (2004). Tissue iron deficiency without anemia impairs adaptation in endurance capacity after aerobic training in previously untrained women. Am. J. Clin. Nutr..

[68] DellaValle D.M., Haas J.D. (2011). Impact of iron depletion without anemia on performance in trained endurance athletes at the beginning of a training season: A study of female collegiate rowers. Int. J. Sport Nutr. Exerc. Metabol..

[69] Dellavalle D.M., Haas J.D. (2014). Iron Supplementation Improves Energetic Efficiency in Iron-Depleted Female Rowers. Med. Sci. Sports Exerc..

[70] Lopez A.M.D., Cacoub P.P., Macdougall I.C.P., Peyrin-Biroulet L.P. (2015). Iron deficiency anaemia. Lancet.

[71] Weiss G. (1999). Iron and anaemia of chronic disease. Kidney Intern..

[72] Malczewska-Lenczowska J., Stupnicki R., Szczepańska B. (2009). Prevalence of iron deficiency in male elite athletes. Biomed. Hum. Kinet..

[73] Constantini N.W., Eliakim A., Zigel L., Yaaron M., Falk B. (2000). Iron status of highly active adolescents: Evidence of depleted iron stores in gymnasts. Int. J. Sport Nutr. Exerc. Metabol..

[74] Nemeth E., Tuttle M.S., Powelson J., Vaughn M.D., Donovan A., Ward D.M.V., Ganz T., Kaplan J. (2004). Hepcidin Regulates Cellular Iron Efflux by Binding to Ferroportin and Inducing Its Internalization. Science.

[75] Ganz T. (2011). Hepcidin and iron regulation, 10 years later. Blood.

[76] Nicolas G., Chauvet C., Viatte L., Danan J.L., Bigard X., Devaux I., Beaumont C., Kahn A., Vaulont S. (2002). The gene encoding the iron regulatory peptide hepcidin is regulated by anemia, hypoxia, and inflammation. J. Clin. Investig..

[77] Moretti D., Goede J.S., Zeder C., Jiskra M., Chatzinakou V., Tjalsma H., Melse-Boonstra A., Brittenham G., Swinkels D.W., Zimmermann M.B. (2015). Oral iron supplements increase hepcidin and decrease iron absorption from daily or twice-daily doses in iron-depleted young women. Blood.

[78] Ganz T., Nemeth E. (2015). Iron homeostasis in host defence and inflammation. Nat. Rev. Immunol..

[79] Schmidt P.J. (2015). Regulation of Iron Metabolism by Hepcidin under Conditions of Inflammation. J. Biol. Chem..

[80] Mccormick R., Moretti D., Mckay A.K.A., Laarakkers C.M., Vanswelm R., Trinder D., Cox G.R., Zimmerman M.B., Sim M., Goodman C. (2019). The Impact of Morning versus Afternoon Exercise on Iron Absorption in Athletes. Med. Sci. Sports Exerc..

[81] Barba-Moreno L., Alfaro-Magallanes V.M., de Jonge X.A.J., Díaz A.E., Cupeiro R., Peinado A.B. (2020). Hepcidin and interleukin-6 responses to endurance exercise over the menstrual cycle. Eur. J. Sport Sci..

[82] Hennigar S.R., McClung J.P., Hatch-McChesney A., Allen J.T., Wilson M.A., Carrigan C.T., Murphy N.E., Teien H.K., Martini S., Gwin J. (2021). Energy deficit increases hepcidin and exacerbates declines in dietary iron absorption following strenuous physical activity: A randomized-controlled cross-over trial. Am. J. Clin. Nutr..

[83] Nemeth E., Rivera S., Gabayan V., Keller C., Taudorf S., Pedersen B.K., Ganz T. (2004). IL-6 mediates hypoferremia of inflammation by inducing the synthesis of the iron regulatory hormone hepcidin. J. Clin. Investig..

[84] Peeling P., Sim M., Badenhorst C.E., Dawson B., Govus A.D., Abbiss C.R., Swinkels D.W., Trinder D. (2014). Iron status and the acute post-exercise hepcidin response in athletes. PLoS ONE.

[85] Galetti V., Stoffel N.U., Sieber C., Zeder C., Moretti D., Zimmermann M.B. (2021). Threshold ferritin and hepcidin concentrations indicating early iron deficiency in young women based on upregulation of iron absorption. eClinicalMedicine.

[86] Badenhorst C.E., Dawson B., Cox G.R., Laarakkers C.M., Swinkels D.W., Peeling P. (2015). Acute dietary carbohydrate manipulation and the subsequent inflammatory and hepcidin responses to exercise. Eur. J. Appl. Physiol..

[87] McKay A.K.A., Peeling P., Pyne D.B., Tee N., Whitfield J., Sharma A.P., Heikura I.A., Burke L.M. (2021). Six Days of Low Carbohydrate, Not Energy Availability, Alters the Iron and Immune Response to Exercise in Elite Athletes. Med. Sci. Sports Exerc.

[88] Fensham N.C., McKay A.K.A., Tee N., Lundy B., Anderson B., Morabito A., Ross M.L., Burke L.M. (2021). Sequential Submaximal Training in Elite Male Rowers Does Not Result in Amplified Increases in Interleukin-6 or Hepcidin. Int. J. Sport Nutr. Exerc. Metabol..

[89] Sim M., Dawson B., Landers G., Swinkels D.W., Tjalsma H., Trinder D., Peeling P. (2013). Effect of exercise modality and intensity on post-exercise interleukin-6 and hepcidin levels. Int. J. Sport Nutr. Exerc. Metabol..

[90] McPherson A.M., Schrader J.W., Docherty C.L. (2019). Ground Reaction Forces in Ballet Differences Resulting from Footwear and Jump Conditions. J. Danc. Med. Sci..

[91] Shaskey D.J., Green G.A. (2000). Sports Haematology. Sports Med..

[92] Ganz T., Olbina G., Girelli D., Nemeth E., Westerman M. (2008). Immunoassay for human serum hepcidin. Blood.

[93] Busbridge M., Griffiths C., Ashby D., Gale D., Jayantha A., Sanwaiya A., Chapman R.S. (2009). Development of a novel immunoassay for the iron regulatory peptide hepcidin. Br. J. Biomed. Sci..

[94] Kroot J.J.C., Hendriks J.C.M., Laarakkers C.M.M., Klaver S.M., Kemna E.H.J.M., Tjalsma H., Swinkels D.W. (2009). (Pre)analytical imprecision, between-subject variability, and daily variations in serum and urine hepcidin: Implications for clinical studies. Anal. Biochem..

[95] Schaap C.C., Heniks J.C.M., Kortman G.A.M., Klaver S.M., Kroot J.J.C., Laarakkers J.M.M., Wiegerinck E.T., Tjalsma H., Janssen M.C., Swinkels D.W. (2013). Diurnal Rhythm rather than Dietary Iron Mediates Daily Hepcidin Variations. Clin. Chem..

[96] Harvey L.J., Armah C.N., Dainty J.R., Foxall R.J., Lewis D.J., Langford N.J., Fairweather-Tait S.J. (2005). Impact of menstrual blood loss and diet on iron deficiency among women in the UK. Br. J. Nutr..

[97] Mayer C., Barker M.K., Dirk P., Moore K.M., McCrudden E., Karakochuk C.D. (2020). Menstrual blood losses and body mass index are associated with serum ferritin concentrations among female varsity athletes. Appl. Physiol. Nutr. Metabol..

[98] Wang W., Bourgeois T., Klima J., Berlan E.D., Fischer A.N., O’Brien S.H. (2013). Iron deficiency and fatigue in adolescent females with heavy menstrual bleeding. Haemoph. Off. J. World Fed. Hemoph..

[99] Bruinvels G., Burden R., Brown N., Richards T., Pedlar C. (2016). The prevalence and impact of heavy menstrual bleeding (Menorrhagia) in elite and non-elite athletes. PLoS ONE.

[100] Dugan C., Scott C., Abeysiri S., Baikady R.R., Richards T. (2021). The need to screen for anemia in exercising women. Medicine.

[101] Rowland T. (2012). Iron Deficiency in Athletes: An Update. Am. J. Lifestyle Med..

[102] De Souza M.J., Toombs R.J., Scheid J.L., O’Donnell E., West S.L., Williams N.I. (2010). High prevalence of subtle and severe menstrual disturbances in exercising women: Confirmation using daily hormone measures. Hum. Reprod..

[103] Petkus D.L., Murray-Kolb L.E., Scott S.P., Southmayd E.A., De Souza M.J. (2019). Iron status at opposite ends of the menstrual function spectrum. J. Trace Elem. Med. Biol..

[104] Torstveit M.K., Sundgot-Borgen J. (2005). Participation in leanness sports but not training volume is associated with menstrual dysfunction: A national survey of 1276 elite athletes and controls. Br. J. Sports Med..

[105] Hincapié C.A., Cassidy J.D. (2010). Disordered Eating, Menstrual Disturbances, and Low Bone Mineral Density in Dancers: A Systematic Review. Arch. Phys. Med. Rehab..

[106] Wilson C., McClung J.P., Philip Karl J., Brothers M.D. (2011). Iron status of military personnel deployed to Afghanistan. Mil. Med..

[107] Diaz M., Rosado J.L., Allen L.H., Abrams S., García O.P. (2003). The efficacy of a local ascorbic acid–rich food in improving iron absorption from Mexican diets: A field study using stable isotopes. Am. J. Clin. Nutr..

[108] Haider L.M., Schwingshackl L., Hoffmann G., Ekmekcioglu C. (2018). The effect of vegetarian diets on iron status in adults: A systematic review and meta-analysis. Crit. Rev. Food Sci. Nutr..

[109] Brown D.D. (2018). Nutritional considerations for the vegetarian and vegan dancer. J. Danc. Med. Sci..

[110] Cialdella-Kam L., Kulpins D., Manore M.M. (2016). Vegetarian, Gluten-Free, and Energy Restricted Diets in Female Athletes. Sports.

[111] Kennedy E.T., Bowman S.A., Spence J.T., Freedman M., King J. (2001). Popular Diets: Correlation to Health, Nutrition, and Obesity. J. Am. Diet. Assoc..

[112] Kenneth V., Shawn H. (2021). Update on vegetarian and vegan athletes: A review. J. Phys. Fit. Sports Med..

[113] Lagowska K., Kapczuk K., Jeszka J. (2014). Nine-month nutritional intervention improves restoration of menses in young female athletes and ballet dancers. J. Int. Soc. Sports Nutr..

[114] Ishizaki S., Koshimizu T., Yanagisawa K., Akiyama Y., Mekada Y., Shiozawa N., Takahaski N., Yamakawa J., Kawano Y. (2006). Effects of a fixed dietary intake on changes in red blood cell delta-aminolevulinate dehydratase activity and hemolysis. Int. J. Sport Nutr. Exerc. Metabol..

[115] Trumbo P., Yates A.A., Schlicker S., Poos M. (2001). Dietary Reference Intakes: Vitamin A, Vitamin K, Arsenic, Boron, Chromium, Copper, Iodine, Iron, Manganese, Molybdenum, Nickel, Silicon, Vanadium, and Zinc. J. Am. Diet. Assoc..

[116] Stoffel N.U., Zeder C., Brittenham G.M., Moretti D., Zimmermann M.B. (2020). Iron absorption from supplements is greater with alternate day than with consecutive day dosing in iron-deficient anemic women. Haematologica.

[117] McCormick R., Dreyer A., Dawson B., Sim M., Lester L., Goodman C., Peeling P. (2020). The effectiveness of daily and alternate day oral iron supplementation in athletes with suboptimal iron status (part 2). Int. J. Sport Nutr. Exerc. Metabol..

[118] Cancelo-Hidalgo M.J., Castelo-Branco C., Palacios S., Haya-Palazuelos J., Ciria-Recasens M., Manasanch J., Pérez-Edo L. (2013). Tolerability of different oral iron supplements: A systematic review. Curr. Med. Res. Opin..

[119] Zhu Y.I., Haas J.D. (1998). Altered metabolic response of iron-depleted nonanemic women during a 15-km time trial. J. Appl. Physiol..

[120] Friedmann B., Weller E., Mairbaurl H., Bartsch P. (2001). Effects of iron repletion on blood volume and performance capacity in young athletes. Med. Sci. Sports Exerc..

[121] Hinton P.S., Giordano C., Brownlie T., Haas J.D. (2000). Iron supplementation improves endurance after training in iron-depleted, nonanemic women. J. Appl. Physiol..

[122] Fogelholm M., Jaakkola L., Lampisjärvi T. (1992). Effects of iron supplementation in female athletes with low serum ferritin concentration. Int. J. Sports Med..

[123] Klingshirn L.A., Pate R.R., Bourque S.P., Davis J.M., Sargent R.G. (1992). Effect of iron supplementation on endurance capacity in iron-depleted female runners. Med. Sci. Sports Exerc..

[124] Tolkien Z., Stecher L., Mander A.P., Pereira D.I.A., Powell J.J. (2015). Ferrous sulfate supplementation causes significant gastrointestinal side-effects in adults: A systematic review and meta-analysis. PLoS ONE.

[125] Stoffel N.U., Cercamondi C.I., Brittenham G., Zeder C., Geurts-Moespot A.J., Swinkels D.W., Moretti D., Zimmermann M.B. (2017). Iron absorption from oral iron supplements given on consecutive versus alternate days and as single morning doses versus twice-daily split dosing in iron-depleted women: Two open-label, randomised controlled trials. Lancet Haematol..

[126] Hall R., Peeling P., Nemeth E., Bergland D., McCluskey W.T.P., Stellingwerff T. (2019). Single versus Split Dose of Iron Optimizes Hemoglobin Mass Gains at 2106 m Altitude. Med. Sci. Sports Exerc..

